# Computational Cardiology: The Heart of the Matter

**DOI:** 10.5402/2012/269680

**Published:** 2012-11-14

**Authors:** Natalia A. Trayanova

**Affiliations:** Department of Biomedical Engineering and Institute for Computational Medicine, Johns Hopkins University, 3400 North Charles Street, Hackerman Hall Room 216, Baltimore, MD 21218, USA

## Abstract

This paper reviews the newest developments in computational cardiology. It focuses on the contribution of cardiac modeling to the development of new therapies as well as the advancement of existing ones for cardiac arrhythmias and pump dysfunction. Reviewed are cardiac modeling efforts aimed at advancing and optimizing existent therapies for cardiac disease (defibrillation, ablation of ventricular tachycardia, and cardiac resynchronization therapy) and at suggesting novel treatments, including novel molecular targets, as well as efforts to use cardiac models in stratification of patients likely to benefit from a given therapy, and the use of models in diagnostic procedures.

## 1. Introduction

The iterative interaction between experimentation and simulation has long played a central role in the advancement of biological sciences. Among computational models of the various physiological systems, the heart is the most highly advanced example of a virtual organ, capable of integrating data at multiple scales, from genes to the whole organ [1]. State-of-the-art whole-heart models of electrophysiology and electromechanics are currently being used to study a wide range of mechanisms in the workings of the normal and the diseased heart [2, 3]. The focus of this paper is on the contribution of heart computational models to the treatment of the diseased heart, that is, on the computational medicine aspect of cardiac modeling applications. Reviewed below are cardiac modeling efforts aimed at advancing and optimizing existent therapies for cardiac disease and at suggesting novel treatments, including novel molecular targets, as well as efforts to use cardiac models in stratification of patients likely to benefit from a given therapy, and the use of models in diagnostic procedures

## 2. Modeling Ventricular Arrhythmias: From Mechanisms to the Clinic

Modeling arrhythmias in the whole heart to reveal mechanisms and suggest better treatments has become one of the most important hallmarks in the utilization of biophysically detailed computational modeling of the heart. A number of ventricular models have focused on arrhythmia dynamics, and specifically on the self-sustained reentrant propagation of complex 3D waves in the ventricles. Historically, these were the first applications of ventricular modeling. Ventricular modeling studies have revealed important aspects of reentrant arrhythmias, among which the dynamic characteristics of ventricular fibrillation (VF), and the role of alternans and restitution in arrhythmogenesis. 

**Figure 1:**
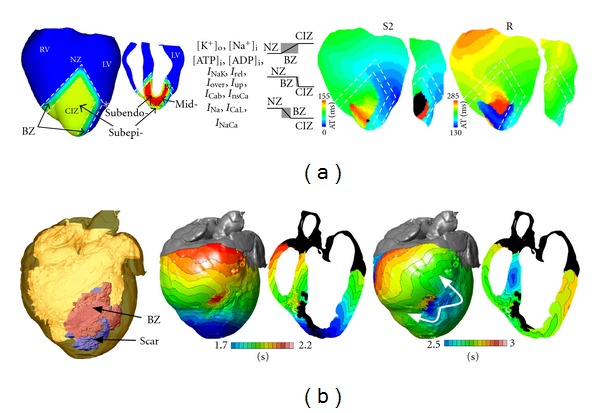
Modeling arrhythmias in ischemia and infarction. (a) Left: model of ischemia phase 1b following LAD occlusion in the rabbit heart, with central ischemic zone (CIZ) and border zone (BZ). Colors distinguish the regions. White dashes outline BZ. Asterisk indicates the stimulus site. Border zones for different ischemic parameters are also shown. Right: generation of reentry in the subepicardium following propagation of premature stimulus in myocardium uncoupled from the surviving endo- and epicardium. Activation maps on the anterior epicardial surface and in a cross-section across the LV are shown. S2 and R refer to activation maps for the premature and the first reentrant beats, respectively. Black asterisk indicates reentry exit site. Figure modified from [15]. (b) Infarct-related VT in the canine heart. Left panel: MRI-based model of the infracted canine heart with scar and BZ. Remaining panels: activation maps of VT following programmed stimulation. Arrows indicate direction of propagation. Figure reprinted from [2].

Ventricular models have been used extensively in characterizing VF dynamics [4, 5]. Particularly interesting are the studies on human hearts [5], which revealed that human VF is driven by a small number of reentrant sources and is thus much more organized than VF in animal hearts of comparable size; the human action potential duration (APD) was found responsible for the specific VF dynamics in the human heart. Furthermore, a number of simulation studies have focused on alternans and their role in arrhythmogenesis. Electrical alternans, which are beat-to-beat changes in APD, have long been recognized as a precursor to the development of VF. Alternans can be concordant with the entire tissue experiencing the same phase of oscillation, or discordant, with opposite-phase regions distributed throughout the tissue. Over the last decade, much emphasis has been placed on the restitution curve slope as a major factor in both the onset of arrhythmias following the development of discordant alternans, and the dynamic destabilization of reentrant waves leading to the transition of ventricular tachycardia (VT) into VF. In what has become known as the restitution hypothesis, flattening the APD restitution curve is postulated to inhibit alternans development and subsequent conduction block, and prevent the onset of VF [6]. Simulation studies employing ventricular models [7–11] have made important contributions to ascertaining the intricate set of mechanisms by and the conditions under which steep APD restitution could lead to VF onset. These include the dynamical transition from concordant to discordant alternans [8], the role of electrotonic and memory effects in suppressing alternans and stabilizing reentrant waves, and the effect of heterogeneous restitution properties on human VF [10, 11]. A recent ventricular modeling study by McDowell et al. [12] employed a novel 3D computational model of the chronically infarcted rabbit ventricles to characterize the arrhythmogenic substrate resulting from myofibroblast infiltration. Furthermore, a number of ventricular simulation studies have incorporated the Purkinje system [13], focusing on the role of the Purkinje system in ventricular arrhythmias. The study by Deo et al. [14] determined the conditions for arrhythmogenesis due to early afterdepolarizations (EADs) originating in Purkinje cells; the latter are known to be more vulnerable to EADs than ventricular myocytes. Finally, simulation of ventricular arrhythmia mechanisms in the setting of regional ischemia [15–17] has characterized the substrate for ischemia phase 1B arrhythmias by examining how the interplay between different degrees of hyperkalemia in the surviving layers, and the level of cellular uncoupling between these layers and the midmyocardium combine with the specific geometry of the ischemic zone in the ventricles to result in reentrant arrhythmias (Figure 1(a), simulation of arrhythmia in a model of regional ischemia in the rabbit ventricles). Computer simulations of cardiac ischemia and the corresponding body surface potentials have also been used to determine how the extent of the ischemic zone is reflected in the 12-lead ECG.

**Figure 2:**
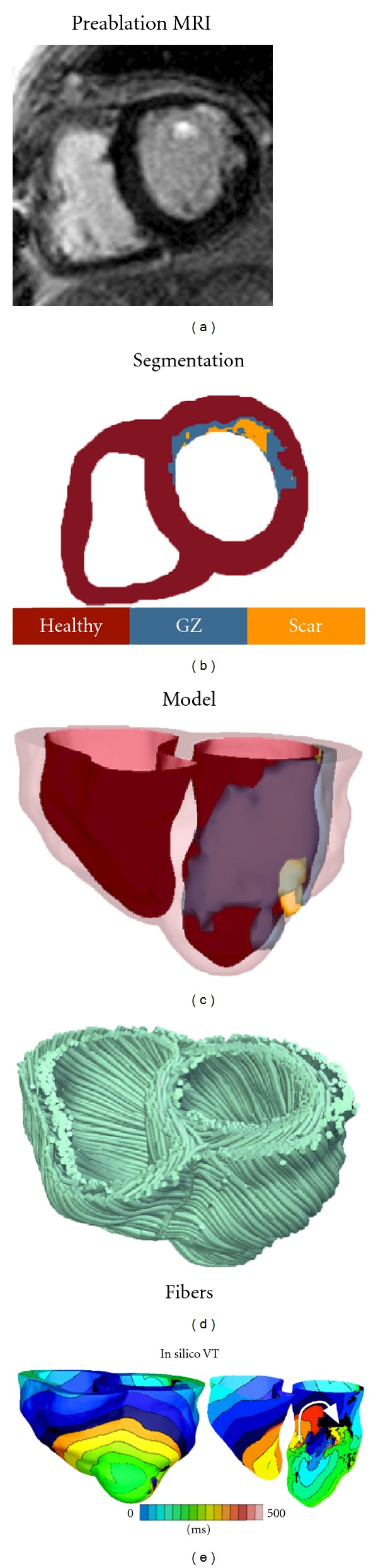
(a), (b) Clinical MRI scan of an infarcted patient heart and the corresponding segmentation. (c) 3D geometric model of the patient heart with the epicardium and the infarct border zone rendered semitransparent. (d) Estimated fiber orientations. (e) Simulated activation map of ventricular tachycardia (VT) revealing reentry on the left ventricular endocardium. VT frequency is 3.05 Hz. Figure modified from [29].

 Recent years have witnessed revolutionary advances in imaging, including ex vivo structural and diffusion tensor (DT) magnetic resonance imaging (MRI) that facilitate acquisition of the intact structure of explanted hearts with high resolution. Leveraging on these advances, a new generation of whole-heart image-based models with unprecedented detail have emerged [18, 19]. Such ex vivo heart models are currently being used, in combination with experimental electrophysiological data, to provide better understanding of the role of the individual morphology of the infarct region in the generation and maintenance of infarct-related VT, the most frequent clinical ventricular arrhythmia, present in 64% of patients with ventricular rhythm disorder and in 89% of patients with sudden cardiac death [20]. A recent MRI-based ventricular modeling study by McDowell et al. [12] employed a 3D computational model of the chronically infarcted rabbit ventricles to characterize the arrhythmogenic substrate resulting from myofibroblast infiltration. It was found that myofibroblasts at low densities do not alter arrhythmia propensity, while at intermediate densities, myofibroblasts cause action potential shortening and exacerbate arrhythmia propensity. Interestingly, at high densities, myofibroblasts were found to protect against arrhythmia by causing resting depolarization and blocking propagation. Using a model of the infracted pig ventricles reconstructed from ex-vivo MRI and DTMRI data, the study by Pop et al. [21] demonstrated a good correspondence between in silico and experimental electroanatomical voltage maps and was able to successfully predict infarct-related VT inducibility after programmed electrical stimulation. Arevalo et al. [22, 23] examined the role of the extent of the infarct border zone in arrhythmogenesis (Figure 1(b), simulation of arrhythmia in MRI-based model of an infracted canine heart), establishing that a minimum volume of remodeled tissue is needed for VT maintenance and demonstrating that the organizing center of infarct-related VT is located within the infarct border zone, regardless of the pacing site from which VT is induced. Such simulation methodology could have a major clinical impact in predicting the optimal targets of catheter ablation of infarct-related VT in individual hearts, should the methodology be able to reconstruct patent hearts from clinical imaging data and evaluate the 3D patterns of infarct-related VT [24] in the patient. The first attempts in this direction have already been made.  Figure 2 presents a simulation of arrhythmia in a patient-specific model of the infracted ventricles; it shows both models generation for the clinical MR scans of the patient heart, as well as the infarct-related ventricular tachycardia. The study by Ng et al. [25] demonstrated the feasibility of using simulations to predict VT circuits. Relan et al. [26] used a hybrid X-ray and MR environment to image a patient heart, which was further personalized with voltage measurements. The results demonstrated that the heart model could successfully be used to assess infract-related VT inducibility from sites not accessible in the clinic. Further translation of ventricular simulations in the clinic will be facilitated by the development of methodologies to estimate patient-specific fiber orientations from clinical MRI scans [27, 28] (Figure 2(d)).

## 3. Modeling Atrial Arrhythmogenesis

Computational modeling of the electrophysiology of the human atria is also becoming an important component in the evaluation and advancement of therapeutic strategies, as recent state-of-the-art biophysically detailed models can accurately simulate the complex spatio-temporal dynamics of atrial arrhythmias. Similar to ventricular modeling methodology, biophysically detailed atrial models are reconstructed from MRI [30] and CT [31] scans, as well as datasets resulting from the Visible Human project [32–34]. Obtaining accurate fiber orientation for the atria, however, has proved more challenging than that in the ventricles, as applying techniques such as diffusion tensor imaging to the atria's thin walls is difficult.

 Atrial models have been used to determine the arrhythmogenicity of substrates due to ionic remodeling [35] or electrophysiological heterogeneity [36]. Modeling studies have also ascertained the role of the autonomic nervous system in atrial arrhythmias, finding that heterogeneous densities of an acetylcholine-driven potassium current lead to a cholinergic form of AF [37]. Atrial modeling has explored the mechanism behind pulmonary vein ectopy, known to initiate AF, finding that activation may arise from differences in resting potential between myocytes and coupled fibroblasts [38]. Other lines of study, pursued with complex atrial models, include representation of intrinsic structure and APD heterogeneity [32, 39–42] and simulated ablation [43–45].

**Figure 3:**
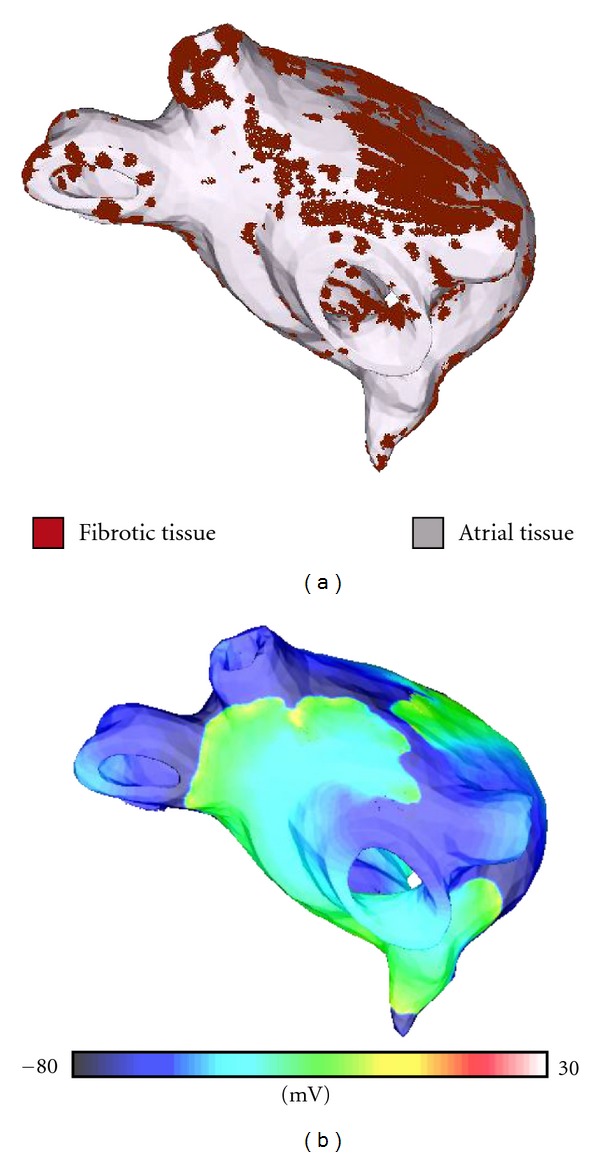
Model of human left atrium with fibrosis (a) and atrial fibrillation in the model (showing transmembrane potential distribution, (b)). Arrhythmia is induced following pulmonary vein ectopy. Figure modified from [53].

 Contemporary atrial models allow for realistic simulation of ECG signals [46], monophasic action potentials [47], and electrograms [48], serving as an important tool for analyzing the etiologies that underlie electrical signals obtained clinically. For example, simulation studies of bipolar electrograms in atrial tissue have found that fibroblast proliferation and microscale obstacles (such as collagenous septa) may be responsible for the fractionation of electrograms [49] during atrial fibrillation (AF), signals which guide catheter ablation of AF. Studies involving simulation of ECG signals in atrial models have helped optimize procedures such as hemodialysis therapy (which can cause AF) [50] and predicting the optimal position and direction of one-channel bipolar ECGs [51]. Full utilization of multiscale atrial models in the clinic for preventative, diagnostic, or therapeutic purposes will require the generation of patient-specific atrial models; early efforts in this direction are already underway [52, 53].  Figure 3 presents the first patient-specific model of AF in the fibrotic atria; panels include the atrial model and the reentrant arrhythmia induced as a result of pulmonary vein ectopy.

## 4. Electromechanical Modeling of Heart Function and Its Application to Cardiac Resynchronization Therapy

**Figure 4:**
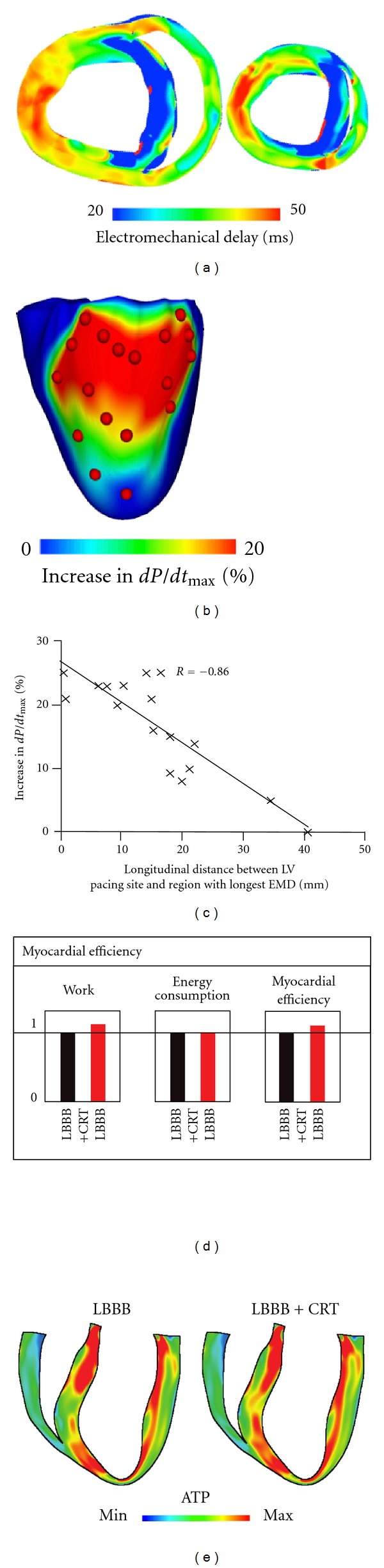
Using electromechanical models of dyssynchronous heart failure to optimize CRT response. (a) Transmural short-axis EMD maps during left bundle branch block (LBBB). (b) Map of the percentage increase in dP/dtmax as a function of the LV pacing site. Red dots denote LV pacing sites. (c) Correlation of longitudinal distance between LV pacing site and region with longest EMD, and percentage increase in dP/dtmax. (d) Bar graph of stroke work (left), total ventricular energy consumption (middle), and myocardial efficiency (right) during LBBB and following CRT. Values are normalized to LBBB (baseline) values. (e) Distribution of ATP consumption during LBBB and following CRT. Figure modified from [54].

Biophysically detailed electromechanical models of the heart, which represent the most sophisticated models of the heart developed thus far [55], have also been harnessed in the development of therapies for pump dysfunction. Cardiac resynchronization therapy (CRT) employs biventricular pacing to recoordinate the contraction of the failing heart. Electromechanical modeling studies have provided insight into the mechanisms that govern CRT efficacy. Kerckhoffs and coworkers [56, 57] have demonstrated that improvement of ventricular function following CRT in the failing heart with left bundle branch block (LBBB) is diminished with increasing infarct size and that infarct location also affects the response to CRT. Niederer et al. [58] revealed that the hemodynamic benefit from CRT is improved when length-dependent tension regulation is attenuated; the study suggested that a compromised Frank-Starling mechanism (the organ-level equivalent of length-dependent tension regulation) could be a clinical metric in identifying heart failure patients as potential responders to CRT. Electromechanical models of the heart have also been used as test beds to understand how different pacing parameters affect CRT efficacy [59, 60]. Constantino et al. [54] recently proposed a strategy to optimize the response to CRT that involves placing the LV pacing electrode at a location that targets the regions with the longest electromechanical delay; maximal hemodynamic benefit occurred when the LV pacing site was located near the base and midventricle, which was within the region of longest electromechanical delay (Figures 4(a), 4(b), and 4(c)). Optimization based on ATP consumption was also considered (Figures 4(d) and 4(e)). Niederer et al. [61] examined whether multisite CRT with a quadripolar lead could result in improved CRT response in a model of human electromechanics; it was found that that multisite CRT conferred a greater hemodynamic improvement only in infarcted hearts. Finally, patient-specific models of hearts with contractile dyssynchrony have been recently developed [62, 63], holding high promise to become an important clinical tool in the treatment of dyssynchronous heart failure.

 With the advancements of computational techniques and medical imaging and image processing, the groundwork for patient-specific modeling of electromechanics has also been laid. Similar to electrophysiological applications as described above, the first step in conducting patient-specific cardiac electromechanical simulations is to construct the computational model of the heart from the patient's medical images. In addition to the challenges in reconstructing heart geometry from in vivo clinical scans of low resolution for any other applications, a specific hurdle in constructing the geometric mesh of a patient's heart for electromechanical applications is defining the unloaded state of the heart, since the heart is constantly loaded during image acquisition. Aguado-Sierra et al. [62] used an iterative estimation scheme to approximate the unloaded geometry from the end-diastolic geometry and ventricular pressures. To help accelerate the clinical adoption of personalized simulations, Lamata et al. [64] developed a robust method that uses variation warping technique to accurately and quickly construct patient-specific meshes from a template heart in a matter of minutes. Equally as important is parameterizing and enriching the electromechanical model of the heart with the patient's own clinical data, as done in studies by Sermesant et al. [63] and Aguado-Sierra et al. [62]. These initial results in the development of patient-specific cardiac electromechanical models attest to the potential utility of cardiac simulation in the diagnosis and treatment of disorders in the pumping function of the heart.

## 5. Computational Modeling of Cardiac Defibrillation 

**Figure 5:**
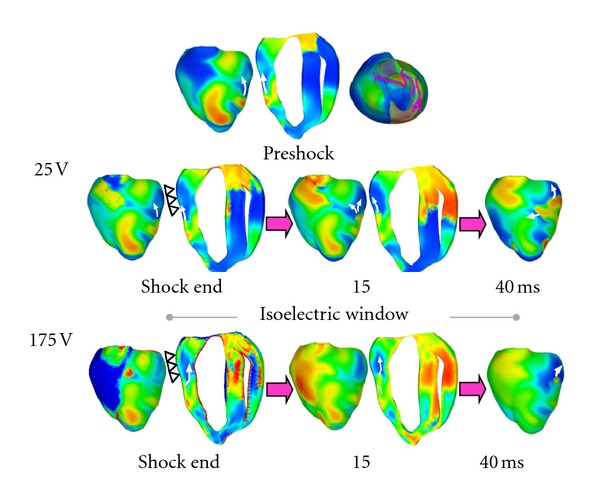
Tunnel propagation of activation following defibrillation shocks in the rabbit heart. Arrows indicate direction of propagation. Presented is the sub- merging of a preshock fibrillatory wavefront by a strong biphasic shock delivered from an ICD. Figure shows the model, the fibrillatory preshock state (with scroll-wave filaments, the organizing centers of reentry, shown in pink), and postshock transmembrane potential maps for two shock strengths at different postshock timings. In contrast to the 25-V shock, the near-DFT 175-V shock converted the left ventricular (LV) excitable area into an intramural excitable tunnel (see triangular arrows in shock-end panel) with no apparent propagation on the epicardium; the wavefront propagated in it until epicardial breakthrough following the isoelectric window. Images based on figures published in [65].

Controlling the complex spatiotemporal dynamics underlying life-threatening cardiac arrhythmias such as fibrillation is extremely difficult because of the nonlinear interaction of excitation waves in a heterogeneous anatomical substrate. In the absence of a better strategy, strong electrical shocks have remained the only reliable treatment for cardiac fibrillation. Over the years, biophysically detailed multiscale models of defibrillation have made major contributions to understanding how defibrillation shocks used in clinical practice interact with cardiac tissue [67–76]. Computer modeling of whole-heart defibrillation has been instrumental in the development of the virtual electrode polarization (VEP) theory for defibrillation. Research has found that mechanisms for shock success or failure are multifactorial, depending mainly on the postshock distribution of transmembrane potential as well as timing and speed of propagation of the shock-induced wavefronts. Recent simulation studies have been instrumental in understanding the mechanisms for the existence of the isoelectric window following defibrillation shocks of strength near the defibrillation threshold (DFT); the isoelectric window is due to propagation of the postshock activations through intramural excitable areas (“tunnel propagation”), bounded by the long-lasting postshock depolarization of the surfaces [65, 77].  Figure 5 presents a simulation of tunnel propagation following the delivery of a defibrillating shock to the fibrillating rabbit ventricles. Simulations have also contributed to understanding of the process of defibrillation in hears with myocardial ischemia and infarction [78–80]. Finally, using a rabbit ventricular electromechanics model, Trayanova et al. [81] conducted simulations of vulnerability to strong shocks and defibrillation under the conditions of LV dilation and determined the mechanisms by which mechanical deformation may lead to increased vulnerability and elevated DFT. The results of the study suggested that ventricular geometry and the rearrangement of fiber architecture in the deformed ventricles are responsible for the reduced defibrillation efficacy in the dilated ventricles.

 Recently, defibrillation modeling has focused on the development of new methodologies for low-voltage termination of lethal arrhythmias or for applying defibrillation in novel, less damaging ways. The study by Tandri et al. [82] was based on the premise that sustained kilohertz-range alternating current (AC) fields have been known to instantaneously and reversibly block electrical conduction in nerve tissue. The article provided proof of the concept that electric fields, such as those used for neural block, when applied to cardiac tissue, similarly produce reversible block of cardiac impulse propagation and lead to successful defibrillation, and that this methodology could potentially be safer means for termination of life-threatening reentrant arrhythmias. The data revealed a previously unrecognized capacity for myocardial cells to be placed in an extended, yet immediately reversible, state of refractoriness by an applied electric field. The imposed refractory state blocked all wave propagation and resulted in termination of reentrant arrhythmias, without impairment of subsequent cellular electrical function or initiation of postshock fibrillatory activity. Since the same AC fields block equally well both neural and cardiac activity, the proposed defibrillation methodology could possibly be utilized to achieve high-voltage yet painless defibrillation.

## 6. Computational Modeling of the Heart as a Testbed for New Molecular Therapies

A major avenue of scientific inquiry in computational cardiology relates the binding/unbinding of drugs to molecular target(s) to the instigation, termination or prevention of cardiac arrhythmias. In this section we focus on examples where emergent behavior, resulting from integrations across the scales of biological hierarchy, has shed new light on existing or novel drug actions for treatment of arrhythmia.

 At the level of the ion channel, Markov models with state specific drug binding/unbinding have been used to test hypotheses regarding the mechanisms of drug effects on macroscopic currents. Since the arrhythmogenic long QT syndrome (LQTS) type 2 is characterized by loss of repolarizing rapid delayed rectifier K^+^ current, *I*_Kr_ [83], a straightforward approach for prevention of LQT-2 arrhythmia, therefore, would be pharmacological enhancement of *I*_Kr_. Perry et al. [84] explored the novel compound RPR260243, shown to enhance *I*_Kr_, represented by the KCNH2 isoform 1a current expressed in *xenopus oocytes*. Rate constants in a proposed modal gating scheme were determined to best fit the experimental data. The model revealed that *I*_Kr_ enhancement could be explained by dose-dependent loss of deactivation; point mutation analysis provided the structural mechanism behind this model prediction. Going a step further, Sale et al. [85] simulated the effects of the drug E4031, known to block *I*_Kr_ in its open state in a use dependent manner [86] on the human ventricular action potential. Drug E4031 is known to block *I*_Kr_ in its open state, in a use-dependent manner [86]. Previously it was unclear why isoform 1a current, which is smaller than 1a/1b combination, was more sensitive to E4031. The Markov model for 1a alone versus 1a/1b mechanistically explained the discrepancy, and related action potential prolongation with E4031 as seen experimentally.

 Type 3 LQTS is brought on by enhancement of the noninactivating, or late Na^+^ current (*I*_Na_) [83]. Using Markov models, Clancy et al. [87] compared the effect of two *I*_Na_ blocking drugs, lidocaine and mexiletine, revealing that mexiletine preferentially binds to the population of channels undergoing late burst opening; the latter events are dangerously common in LQT3, leading to arrhythmogenic early afterdepolarizations (EADs). By contrast, lidocaine preferentially binds to channels during the rapid activation/inactivation phase of *I*_Na_. The model showed that there are mexiletine doses, which selectively remove late current and EADs without detrimental effect to excitability.

 Nesterenko et al. [88] also drew connections between drug binding kinetics and emergent effects on the AP in the investigation of the novel antiarrhythmic drug ranolazine. Among its many targets [89], ranolazine reduces late *I*_Na_ in atrial-selective fashion [90]; Nesterenko et al. explained the mechanism behind this selectivity by introducing the concept of a “pre-open” state to the *I*_Na_ Markov model. The effect of late *I*_Na_ block by ranolazine on tissue electrophysiology was examined by Morita et al. [91], who demonstrated that late *I*_Na_ block suppresses EADs that lead to focal reentry after hydrogen peroxide application, as observed in experiments. Importantly, ranolazine is a mild *I*_Kr_ blocker at clinical doses, an effect of major significance for drug safety. Since current FDA regulations require that new candidate drugs not block *I*_Kr_, ranolazine would not have been approved if discovered today. Rodriguez et al. [92] proposed that computer modeling of multichannel affecting drugs, such as ranolazine, could be a testbed for determining the utility of new or previously rejected compounds or drug combination approaches, with modeling as a force against rigid standards, and toward rational, more holistic drug candidate selection. Fundamentally, this is the modeling approach of Sarkar and Sobie [93] whose recent article explores basic mechanisms by which interrelated model parameters contribute to the consequence of *I*_Kr_ block, a phenomenon known as the “repolarization reserve” [94]. The article makes the important discovery that subtle changes in ion channel substrate can have profound and indirect effects on the response to drugs. Simulating the subtle differences between species [95] and the effects of sex hormones [96, 97] also demonstrated changes in drug block effects. Personalized medicine requires clear delineation of the subtle interspecies and interindividual differences, which determine outcomes; this delineation is made possible in part by mechanistic simulation. 

**Figure 6:**
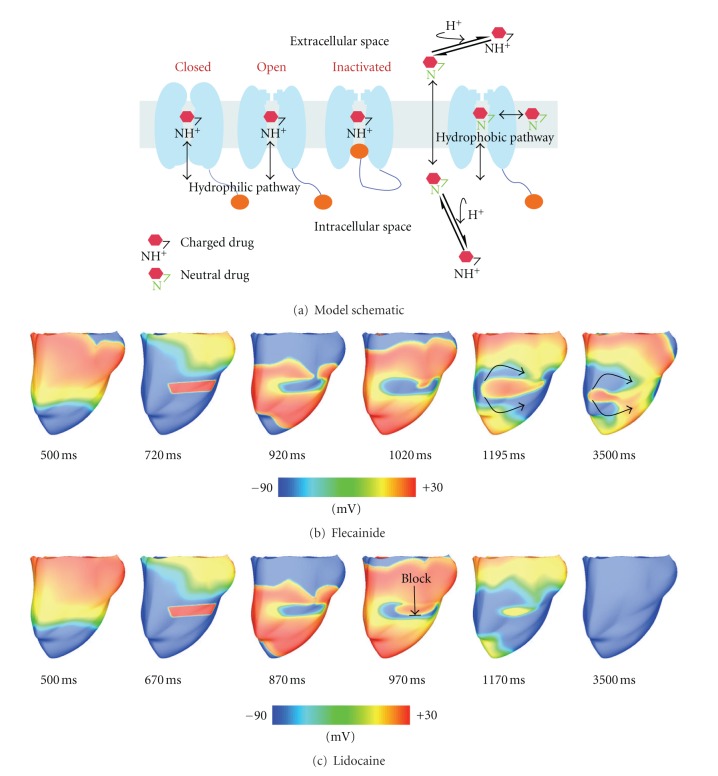
Simulation of drug-related arrhythmias. (a) Schematic for drug binding to sodium channels. Maps of the phase variable in (b), sustained figure-of-eight reentry with 2 *μ*M flecainide, and (c) nonsustained reentry with 20 *μ*M lidocaine, following premature stimuli (S2). Sustained reentry occurred when applying S2 within the vulnerable window (VW) of the model with 2 *μ*M flecainide (VW = 660 ms–735 ms), but not for the model with 20 *μ*M lidocaine (VW = 630 ms–685 ms), as indicated by the 970 ms time point of (c). Figure modified from [66].

 Relating effects of drugs on ion channels beyond the AP require virtual tissue or whole heart organ simulation, to examine arrhythmia onset, termination, and prevention. Recently, Benson et al. [98] related the effects of d-sotalol, an *I*_Kr_ blocker, and amiodarone, a complex multichannel effector, to arrhythmia formation in the heterogenous canine ventricular wedge. An emergent finding was the understanding of how the vulnerability window is enhanced by d-sotalol but reduced by amiodarone due to different effects of the drugs on different cell types. Whereas the drug models used by Benson et al. were implemented by simple conduction scaling, a new study by Moreno et al. has incorporated both state-dependent Markov modeling of drug effects and full integration to the human AP, human tissue, and finally realistic MRI image-based human heart [66]. This is the first instance of such massive integration across the space and time scales at play. Moreno et al. showed that the effects of flecainide and lidocaine on *I*_Na_ block are globally similar in response to dynamic protocols. However, clinical trials have shown previously that flecainide tended to be proarrhythmic at therapeutic doses, while lidocaine was not. Moreno et al. results make clear that neither simple reduction in sodium conductance nor single cell simulation can resolve this paradox. At the macroscopic scale, the vulnerable window was greater for flecainide than for lidocaine (especially in heart failure simulations due to shortened diastole) and reentrant arrhythmia in the ventricle persisted (Figure 6). At the microscopic scale, Markov models explained that this was due to the relatively slow accumulation of and recovery from use dependent block with flecainide. 

## 7. Noninvasive Electroanatomic Mapping of the Heart

The electrocardiogram (ECG), a century-old tool for assessing the electrical activity of the heart, is a global manifestation, on the body surface, of the electrical activity that takes place in the heart during the cardiac cycle. The electrical sources within the heart, separated by tissues from the body surface, are sampled on the body surface by only a few electrodes. This poses a challenge in the treatment of lethal ventricular arrhythmias. Thus, clinicians have sought to gain insight into the spatiotemporal patterns of electrical activation in the heart by interpreting other noninvasively recorded signals. Inverse electrocardiography represents the development on noninvasive methodology for assessing the spatiotemporal electrical activation in the heart, in which body surface electrograms and patient-specific anatomical data are combined with state-of-the-art computational techniques. From a mathematical standpoint, the reconstruction of signals in the domain of the heart from body surface measurements is ill-posed, in that it can easily be corrupted by low-amplitude electric noise or minute positional errors. Thus, the development of tools that can be implemented in clinical practice has been a long and arduous road.

 The application of inverse electrocardiography in humans has been led by the Rudy lab, whose electrocardiographic imaging (ECGI) method computes epicardial extracellular potential distributions from body surface potential maps. ECGI has been performed on Wolff-Parkinson-White (WPW) patients before and after accessory pathway ablation [99], to characterize the size and extent of scar in postmyocardial infarction patients [100], to identify responders to CRT therapy [101], to noninvasively map infarct-related ventricular arrhythmias [102], and to examine the spontaneous initiation of ventricular tachycardia (VT) and its termination with antitachycardia pacing. The technique was also used in a wide variety of AF patients and has demonstrated that multiple AF mechanisms (isthmus propagation, macroscopic reentry, multiple coexisting wavelets, and wave break) often occur simultaneously in the same patient [103].

 Studies by the teams of He, Oosterom, Berger, and Kalinin [104–108] have advanced noninvasive electrocardiographic imaging beyond the mapping of epicardial electrograms to mapping intramural or endocardial electrical activity. The inverse methodology by the He team reconstructs an equivalent current density field throughout the ventricles, from which 3D maps of activation sequence are generated; while it has not been yet utilized in patient studies, the technique was able to accurately pinpoint sources of endocardial ectopy in pigs [109] and to image activation sequences during pacing and VT in both rabbits [110] and dogs [111]. Kalinin's approach [108, 112] to map epicardial and endocardial electrograms has been already applied in 200 patients in Russian hospitals [113]. Similarly, the methodology developed by Berger and coworkers has applied to identify preexcitation sites in WPW patients [107] and to provide detail on simultaneous endocardial and epicardial activation sequences during CRT [105]. 

## 8. Biophysically Detailed Computational Modeling of the Heart in Risk Stratification for Arrhythmias

Robust methods for stratifying the risk of lethal cardiac arrhythmias decrease morbidity and mortality in patients with cardiovascular disease and reduce health care costs [114]. The most widely used approaches currently used for stratifying risk of cardiac arrhythmias involve testing for abnormalities in the ECG, then using the results to identify patients who would benefit from ICD therapy. ECG-based risk stratification methods scan for abnormalities in ventricular depolarization (late potentials [115] and a fractionated QRS complex [116]) and repolarization (T-wave alternans [117], QT variability or dispersion [118, 119]). However, the mechanisms underlying these ECG indices, and their relationship to lethal cardiac arrhythmias, are not fully understood. This lack of knowledge likely explains why results of clinical trials to correlate surface EGG indices to lethal cardiac arrhythmias are often contradictory [114]. Computational models of the heart have made inroads in this clinical cardiology arena [98, 120–126].

Research has reported a strong correlation between increased arrhythmia risk and the presence of T-wave alternans [127, 128]. In the clinical setting, testing for Microvolt T-wave Alternans (MTWA) has particularly shown promise for dichotomizing patients that would and would not benefit from ICD therapy [129, 130]. However, the mechanistic basis of MTWA preceding lethal ventricular arrhythmias has been under debate. Until recently, it was believed that a steep action potential duration (APD) restitution (>1) at rapid heart rates [131] produces alternans in APD that underlie T-wave alternans and the genesis of fibrillation [132]. However, MTWA is most successful in stratifying risk in patients at heart rates <110 bpm, where APD restitution is flat [133]. Computational models of the LV wall in combination with clinical data revealed that abnormal handing of intracellular calcium underlies alternans in action potential voltage, which result in MTWA at heart rates <110 bpm [120, 121]; abnormalities in intracellular calcium have long been linked to ventricular fibrillation [134, 135]. Computational modeling studies have also shown that under the conditions of abnormal calcium dynamics, the magnitude of the T-wave alternans is enhanced by structural heterogeneities in the myocardium [122].

A recent study used MRI-based computational model of the human ventricles to demonstrate that detecting instabilities in the QT interval in the clinical ECGs can predict the onset of VT, particularly in patients with acute myocardial infarction [126]. By having the ability to easily control the frequency and degree of premature activations in the model, the studies found that increased frequency of premature activation can precede the onset of VT, with the premature activations placing the system in a state where QT interval is unstable. Therefore, screening the QT interval of the ECG for instabilities using the novel algorithm developed by Chen and Trayanova [126, 136] could potentially be a robust risk stratification method for patients with acute myocardial infarction. These study pave the way for executing computer simulations to determine patient-specific thresholds for arrhythmia stratification ECG indices, rather than relaying on clinical guidelines based on large and diverse cohorts of patients. Another approach for stratifying the risk of lethal cardiac arrhythmias that has recently gained traction is the use of computer models to predict the arrhythmia outcome in patients that exhibit potentially lethal mutations in genes encoding cardiac proteins associated with the long-QT syndrome [98, 123–125]. These studies chart new directions for future genotype-based risk stratification and personalized gene therapy.

## 9. Final Words

Modern cardiac research has increasingly recognized that computational models of the heart can help interpret an array of experimental data and dissect important mechanisms and relationships. This paper focused on another aspect of computational modeling of the heart: its translational potential. Computer simulations of the function of the diseased heart represent a profound example of a research avenue in the new discipline of computational medicine [29], aiming specifically at improving the clinical practice of cardiology. Biophysically detailed models of the heart assembled with data from cardiac imaging modalities that incorporate electromechanical and structural remodeling in cardiac disease are well poised to become a first line of screening of new therapies and approaches, new diagnostic developments, and new methods for disease prevention. Implementing patient-specific cardiac simulations at the patient bedsides could become one of the most thrilling examples of multidisciplinary approaches in translational medicine.
